# Intraovarian Platelet-Rich Plasma for Women with Diminished Ovarian Reserve: A Systematic Review and Meta-Analysis

**DOI:** 10.3390/jcm15072482

**Published:** 2026-03-24

**Authors:** Xinyi Wang, Hongyi Wei, Xi Du, Haojie He, Caihong Ma

**Affiliations:** 1 State Key Laboratory of Female Fertility Promotion, Center for Reproductive Medicine, Department of Obstetrics and Gynecology, Peking University Third Hospital, Beijing 100191, China; wangxydoct@163.com (X.W.);; 2 National Clinical Research Center for Obstetrics and Gynecology, Peking University Third Hospital, Beijing 100191, China; 3 Key Laboratory of Assisted Reproduction, Peking University, Ministry of Education, Beijing 100191, China; 4 Beijing Key Laboratory of Collaborative Innovation in Frontier Technologies for Population Quality, Beijing 100191, China; 5 National Clinical Key Specialty Construction Program, Beijing 100191, China; 6 Department of Obstetrics and Gynecology, Peking University International Hospital, Beijing 102206, China; 13811252576@126.com; 7 Department of Obstetrics and Gynecology, Peking University Third Hospital, Beijing 100191, China

**Keywords:** platelet-rich plasma, diminished ovarian reserve, premature ovarian insufficiency, poor ovarian response

## Abstract

**Objectives:** To systematically evaluate the efficacy and safety of intraovarian platelet-rich plasma (PRP) administration in women with diminished ovarian reserve (DOR) and related conditions, given the growing clinical interest and the conflicting evidence from uncontrolled and controlled studies. **Methods:** This systematic review and meta-analysis followed the Preferred Reporting Items for Systematic Reviews and Meta-Analyses (PRISMA) guidelines. Comprehensive searches were performed in PubMed, Web of Science, EMBASE, the Cochrane Central Register of Controlled Trials (CENTRAL), and Scopus up to January 2026. Eligible studies included randomized controlled trials (RCTs), prospective cohort studies, and before–after studies investigating PRP-based interventions in women diagnosed with DOR, premature ovarian insufficiency (POI), or poor ovarian response (POR). Given the limited availability of controlled data, these populations were analyzed together with cautious interpretation. Study quality was assessed using the Joanna Briggs Institute (JBI) checklists and the Critical Appraisal Skills Programme (CASP) tool for RCTs. Pooled estimates were calculated using random- or fixed-effects models depending on heterogeneity (I^2^). **Results:** Nineteen studies involving 1794 women were included, of which two were randomized controlled trials. In single-arm and before–after analyses, PRP administration was associated with increases in serum anti-Müllerian hormone (AMH) levels and antral follicle count (AFC), as well as a reduction in serum follicle-stimulating hormone (FSH). In addition, the number of metaphase II (MII) oocytes retrieved and transferable embryos increased following PRP treatment. However, pooled analyses of controlled studies, including RCTs, did not demonstrate consistent improvements in mature oocyte yield compared with control groups. In single-arm analyses, the pooled clinical pregnancy rate and live birth rate following PRP treatment were 15.5% (95% CI: 11.1–21.2%) and 10.7% (95% CI: 6.7–16.6%), respectively. No major procedure-related adverse events were reported across included studies. **Conclusions:** In conclusion, intraovarian PRP is associated with improvements in ovarian reserve markers such as AMH and AFC in uncontrolled studies. However, evidence from randomized controlled trials does not demonstrate a consistent benefit in pregnancy and live birth. Well-designed RCTs with standardized protocols are needed before clinical recommendation.

## 1. Introduction

As the average age of childbearing increases and the prevalence of infertility rises worldwide, impaired ovarian function has become a major challenge in reproductive medicine. Diminished ovarian reserve (DOR) is characterized by a reduced quantity and quality of oocytes relative to chronological age and is commonly associated with suboptimal responses to ovarian stimulation and poor reproductive outcomes. Although related, DOR, premature ovarian insufficiency (POI), and poor ovarian response (POR) represent overlapping but clinically distinct entities with heterogeneous diagnostic criteria and prognoses. It is estimated that approximately 1–3% of women under 40 years of age are affected by POI or its advanced forms [1,2]. Collectively, these conditions are associated with reduced oocyte yield, compromised embryo development, and decreased fertility in both natural conception and assisted reproductive technology (ART) settings, often accompanied by menstrual disturbances and significant psychosocial burden.

To date, therapeutic strategies aimed at improving fertility outcomes in women with DOR remain limited. Hormone replacement therapy (HRT) is primarily used for symptom control and long-term health protection but does not restore ovarian function or fertility and may be associated with hormone-related risks [3]. Growth hormone (GH) supplementation [4], antioxidant therapy (such as vitamins A, C, and E and carotenoids) [5], and mesenchymal stem cell transplantation (including bone marrow, adipose tissue, and menstrual fluid) [6,7,8] have shown potential in experimental or early clinical studies, yet their efficacy and safety remain inconclusive. Although in vitro fertilization and embryo transfer (IVF-ET) are widely applied [9], their success largely depends on the availability of competent oocytes and embryos, which are often limited in DOR patients [10]. Therefore, therapeutic strategies aiming to enhance endogenous ovarian activity are of great clinical significance.

Platelet-rich plasma (PRP), an autologous plasma derivative enriched with platelets and a wide array of growth factors, has recently emerged as a potential regenerative strategy in reproductive medicine. PRP contains bioactive molecules such as platelet-derived growth factor (PDGF), transforming growth factor-β (TGF-β), and vascular endothelial growth factor (VEGF), which may promote angiogenesis, modulate local inflammation, and mitigate oxidative stress within ovarian tissue [11,12]. On this basis, intraovarian PRP administration has been proposed as a means to improve the ovarian microenvironment and potentially stimulate residual follicular activity. Several preliminary studies have reported improvements in surrogate markers of ovarian reserve, including anti-Müllerian hormone (AMH) levels [13] and antral follicle count (AFC) [14,15], as well as increases in oocyte retrieval and pregnancy rates following PRP treatment. However, these findings are predominantly derived from uncontrolled or before–after studies, while controlled trials have yielded inconsistent results, particularly with respect to live birth outcomes. Moreover, substantial heterogeneity in PRP preparation, activation methods, and administration protocols further complicates interpretation of the available evidence.

In light of these uncertainties, a comprehensive synthesis of the existing literature is needed. We therefore conducted a systematic review and meta-analysis to critically evaluate the efficacy and safety of intraovarian PRP administration in women with DOR and related conditions, with a particular focus on distinguishing evidence derived from uncontrolled studies from that obtained in controlled clinical trials. By systematically assessing both ovarian reserve parameters and reproductive outcomes, this study aims to provide a balanced appraisal of the current evidence and to inform future clinical research and trial design.

## 2. Materials and Methods

### 2.1. Protocol and Reporting Standards

This systematic review and meta-analysis was conducted in accordance with the Preferred Reporting Items for Systematic Reviews and Meta-Analyses (PRISMA) statement. The review protocol was prospectively registered in PROSPERO (CRD420251151899). Trial Registration: PROSPERO Identifier: CRD420251151899.

### 2.2. Literature Search

A comprehensive literature search was performed in PubMed, Web of Science, EMBASE, the Cochrane Central Register of Controlled Trials (CENTRAL), and Scopus from database inception to 31 January 2026. Search terms combined Medical Subject Headings (MeSH) and free-text terms related to “platelet-rich plasma”, “PRP”, “diminished ovarian reserve”, “poor ovarian response”, “premature ovarian insufficiency”, and related synonyms. The full search strategies for each database are provided in Table S1. Reference lists of included studies and relevant reviews were screened manually to identify additional eligible studies. The search was restricted to articles published in English.

### 2.3. Eligibility Criteria and Study Selection

Studies were eligible if they met the following criteria: (1) population: adult women (≥18 and ≤50 years, the upper age limit of 50 years was selected to focus on women of reproductive age and to align with the typical age range in clinical studies of ovarian reserve interventions, while excluding perimenopausal or menopausal populations where ovarian rejuvenation strategies may differ fundamentally) diagnosed with diminished ovarian reserve (DOR), premature ovarian insufficiency (POI), or poor ovarian response (POR) according to established criteria (e.g., Bologna [16] or POSEIDON [17]). Given the limited number of studies available in this emerging field and the clinical overlap of these conditions as a spectrum of impaired ovarian function, a broader inclusion criterion was adopted to comprehensively survey the potential effects of intraovarian PRP and to generate preliminary hypotheses. However, the results are interpreted with caution, acknowledging the inherent heterogeneity of these populations; (2) intervention: intraovarian platelet-rich plasma (PRP) injection or PRP-based regimens; (3) comparator: saline, standard care, or pre-treatment baseline in before–after studies; (4) outcomes: at least one of the following—ovarian reserve markers (AMH, AFC, FSH), oocyte/embryology outcomes (MII oocyte count, number of transferable embryos), clinical pregnancy, and live birth; (5) study design: randomized controlled trials (RCTs), prospective cohort studies, or before–after interventional studies. Exclusion criteria included studies involving significant concomitant systemic diseases (e.g., active malignancy), male-factor infertility as the primary cause without separate female outcomes, case reports, reviews, conference abstracts without full data, and non-English publications.

Titles and abstracts retrieved from the searches were screened independently by two reviewers (X.W. and H.W.). Full texts of potentially eligible records were then assessed for inclusion by the same reviewers. Disagreements were resolved by discussion and, when necessary, adjudicated by a third reviewer (Q.D.).

### 2.4. Data Extraction

Two reviewers (X.W. and H.W.) independently extracted data using a pre-piloted form. Extracted data included first author, year of publication, country, study design, sample size, participants’ mean age and BMI, diagnostic criteria for ovarian impairment, PRP preparation method (blood volume, centrifugation protocol, activation method if reported), injection route and volume, follow-up duration, and outcomes of interest (AMH, AFC, FSH, number of MII oocytes, number of transferable embryos, clinical pregnancy, live birth). When necessary, corresponding authors were contacted for missing data. Discrepancies were resolved by consensus.

### 2.5. Quality Assessment

Risk of bias and methodological quality were assessed independently by two reviewers (X.W. and H.W.) using appropriate tools: the Joanna Briggs Institute (JBI) checklists for quasi-experimental/before–after and cohort studies, and the Critical Appraisal Skills Programme (CASP) checklist for randomized controlled trials. The CASP was chosen for its applicability to small RCTs with limited reporting, while key Cochrane domains (randomization, allocation concealment, blinding) were extracted and reported separately.

For RCTs, additional domains such as randomization, allocation concealment, and blinding were recorded. Any disagreements were resolved by discussion or adjudication by a third reviewer.

### 2.6. Statistical Analysis

Continuous outcomes (e.g., AMH, AFC, number of MII oocytes) were pooled as mean differences (MD) with 95% confidence intervals (CIs). Dichotomous outcomes (e.g., clinical pregnancy, live birth) were pooled as event rates with 95% CIs or risk ratios (RRs) where a control group was available. Single-arm pooled rates were used to describe post-intervention outcomes but were not intended to infer treatment efficacy in the absence of a control group. Heterogeneity across studies was assessed using the Cochran’s Q test and quantified with the I^2^ statistic. Given the anticipated clinical and methodological diversity in patient populations, diagnostic criteria, and especially PRP preparation protocols, a random effects (DerSimonian and Laird) model was chosen a priori to provide a more conservative pooled estimate. Sensitivity analyses were performed using a leave-one-out approach (Figures S1 and S2). Where sufficient data were available, subgroup analyses were planned a priori by age group, diagnostic criteria (Bologna vs. POSEIDON), and PRP preparation method. Publication bias was evaluated through visual inspection of funnel plots and Egger’s regression test for the primary outcome with ≥10 studies (Figure S3). All statistical analyses were performed using Review Manager (RevMan) version 5.3 and Comprehensive Meta-Analysis (CMA) version 3.0 (Biostat, Englewood, NJ, USA). A two-sided *p*-value < 0.05 was considered statistically significant.

### 2.7. Ethics Statement

Ethical approval was not required for this systematic review and meta-analysis because all data were obtained from published studies.

## 3. Results

### 3.1. Study Selection and Characteristics

**Figure 1:**
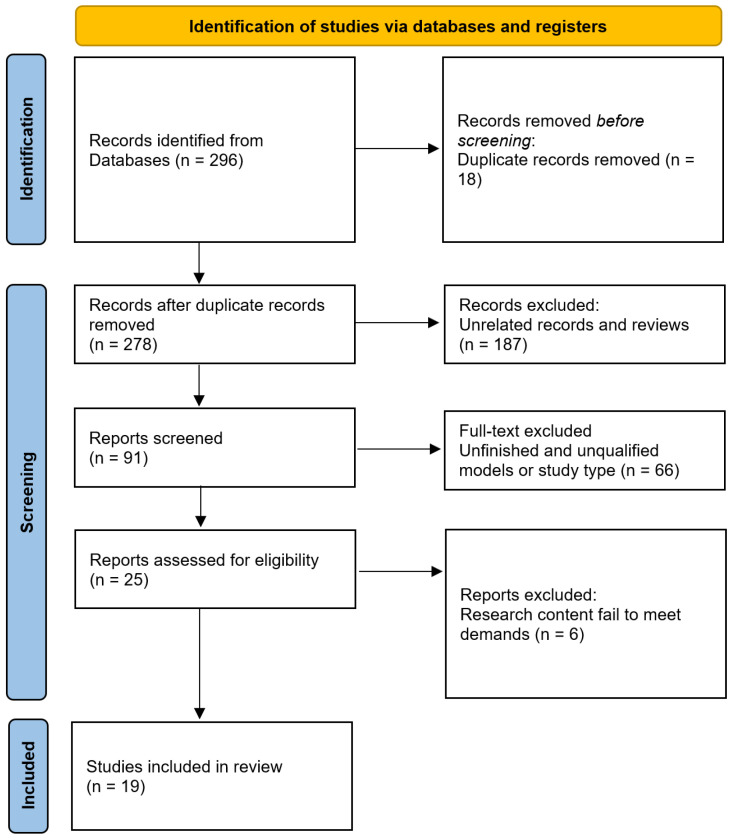
Study flow diagram.

The initial database search identified 296 records, of which 18 were duplicates. After screening titles and abstracts, 253 records were excluded for irrelevance or failure to meet inclusion criteria. The full texts of 25 studies were assessed, and 19 studies involving 1794 women were finally included in the meta-analysis. No unpublished or ongoing trials were identified. A PRISMA flow diagram summarizing the screening process is presented in Figure 1. The PRISMA checklist can be seen in Table S2.

The included studies were published between 2019 and 2026 and were conducted in ten countries (Iran, Turkey, Spain, the USA, Bulgaria, Argentina, Greece, Venezuela, Macedonia, and Ukraine). All were prospective studies, including 2 randomized controlled trials (RCTs), 5 cohort studies, and 12 before–after designs. Sample sizes ranged from 12 to 510 participants, with ages between 18 and 50 years. Seven studies applied the Bologna criteria and four used the POSEIDON classification for diagnosing diminished ovarian reserve. PRP was prepared from autologous peripheral blood and injected into both ovaries under transvaginal ultrasound guidance (Table 1).

### 3.2. Quality Assessment Outcome

Study quality was assessed using the Joanna Briggs Institute (JBI) checklists and the CASP tool for RCTs. Most studies were rated as moderate quality, with clear inclusion criteria and adequate outcome reporting. However, randomization and blinding were insufficiently described in several studies, and the absence of control groups in before–after designs introduced potential bias (Table S3).

### 3.3. Primary Outcomes

In this part, we compared a series of ovarian reserve indicators including FSH, AMH and AFC before and after PRP intervention. Results are presented in the form of forest graphs.

**Figure 2:**
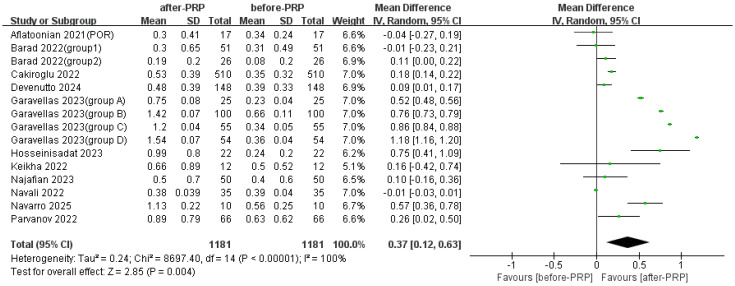
Comparison of AMH before and after PRP [13,14,15,18,19,21,24,25,26,30,31].

Eleven studies evaluated changes in AMH levels before and after PRP injection. The pooled results showed a significant increase (Figure 2).

**Figure 3:**
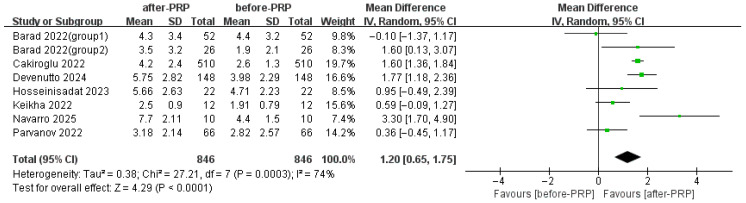
Comparison of AFC before and after PRP [13,14,15,19,21,24,31].

Seven studies assessed AFC, which also increased significantly after PRP (Figure 3).

**Figure 4:**
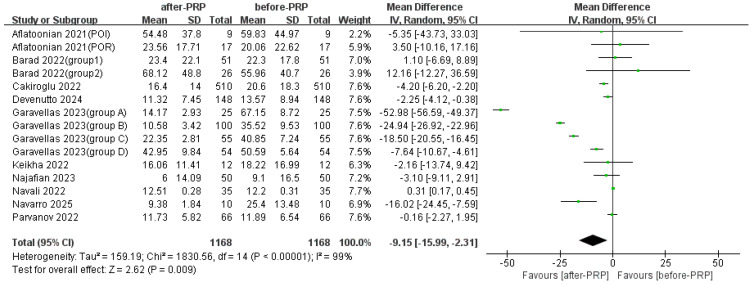
Comparison of FSH before and after PRP [14,15,18,19,21,24,25,26,30,31].

FSH levels were observed to decrease after PRP treatment (Figure 4).

### 3.4. Secondary Outcomes

We collected the number of MII oocytes and transferable embryos, including cleavage stage embryos (D3 and blastocysts), considering their value in offering auxiliary assessment of pregnancy.

**Figure 5:**
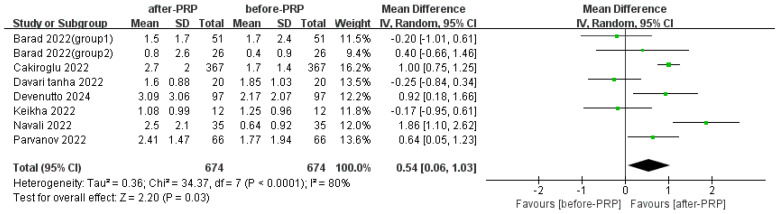
MII oocytes before and after PRP treatment [14,15,19,21,24,26,28].

**Figure 6:**
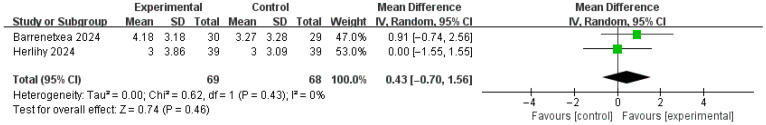
MII oocytes comparison in randomized controlled trials [22,23].

Pooled analysis from seven studies showed a modest increase in the number of MII oocytes after PRP treatment (mean difference = 0.64, 95% CI 0.06–1.03, *p* < 0.0001, I^2^ = 80%) (Figure 5). While for two RCTs, MII oocytes showed no overall difference between the experimental and control groups (Figure 6).

**Figure 7:**
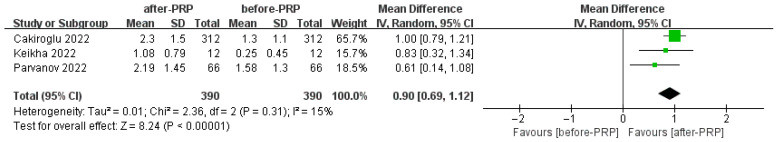
Comparison of transferable embryos before and after PRP [14,19,24].

As for transferable embryos, due to the ambiguous description, statistics of D3 and blastocysts were merged together for analysis. Three studies demonstrated that the number of transferable embryos increased significantly after PRP (Figure 7).

Based on single group rate meta-analysis, clinical pregnancy rate and live birth rate before and after PRP intervention were respectively presented in the forest graphs. In our study, clinical pregnancy was defined as the number of clinically diagnosed pregnant women divided by the total number of PRP subjects. As for the definition of clinically pregnant, either criterion met is consilient:

(a) Detected fetal heart activity in transvaginal ultrasonography 5 weeks after positive beta hCG;
(b) A pregnancy that is confirmed by both high levels of hCG and ultrasound confirmation of a gestational sac or fetal pole.

**Figure 8:**
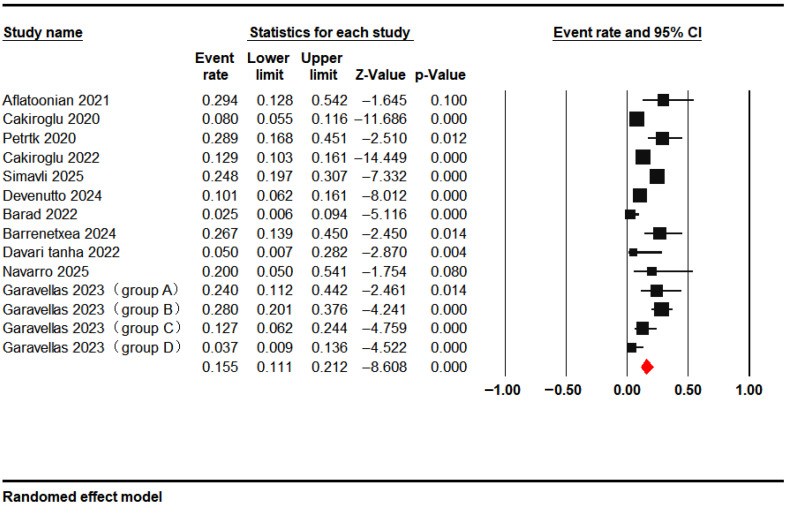
Clinical Pregnancy Rate [14,15,18,20,21,22,27,28,30,31,33].

Eleven studies reported clinical pregnancy outcomes, with a pooled rate of 15.5% (95% CI: 11.1–21.2%) following PRP intervention (Figure 8).

It must be emphasized that the following pooled rates are derived from single-arm analyses and, in the absence of a comparator group, cannot be causally attributed to the PRP intervention. They should be interpreted as descriptive outcomes within the treated population.

Live birth rate is defined as the ratio of the number of patients who finally gave birth to a live child to the number of subjects who received PRP therapy.

**Figure 9:**
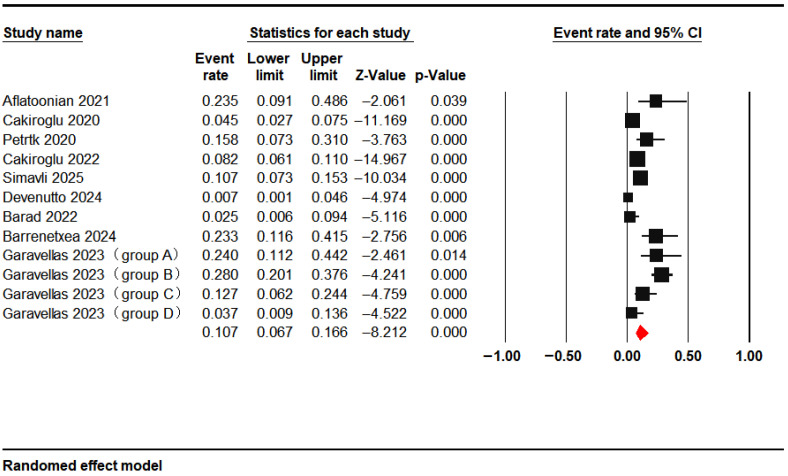
Live Birth Rate [14,15,18,20,21,22,27,30,33].

Nine studies provided live birth data, yielding a pooled live birth rate of 10.7% (95% CI: 6.7–16.6%) (Figure 9).

It is important to note that findings derived from single-arm or before–after studies should be interpreted with caution, as such designs are susceptible to biases like regression to the mean and cannot establish causality.

### 3.5. Sensitivity and Publication Bias

Sensitivity analyses confirmed the stability of the results, and Egger’s test indicated no significant publication bias.

Sensitivity analysis using the leave-one-out method demonstrated that no single study significantly altered the pooled effect size, confirming the robustness of the results. Funnel plot inspection and Egger’s regression test suggested no significant publication bias.

## 4. Discussion

This systematic review and meta-analysis evaluated the efficacy and safety of intraovarian platelet-rich plasma (PRP) administration in women with diminished ovarian reserve (DOR) and related conditions. By synthesizing data from 19 prospective studies involving 1794 participants, our findings indicate that PRP treatment is associated with moderate improvements in certain ovarian reserve markers, notably serum anti-Müllerian hormone (AMH) levels and antral follicle count (AFC). AMH and AFC are direct, quantitative indicators of the ovarian follicular pool and are sensitive to early changes in ovarian function. The observed increases following PRP administration are biologically plausible, likely reflecting an enhanced local ovarian microenvironment—promoted by PRP-derived growth factors—that may support follicular recruitment or survival. In contrast, the absence of a significant reduction in FSH levels is not unexpected. FSH is regulated by systemic endocrine feedback loops and may be less responsive to localized intraovarian interventions over short follow-up periods. This dissociation between local ovarian markers and systemic hormones has been noted in other “ovarian rejuvenation” studies and underscores the complexity of assessing treatment response in DOR [34].

Regarding reproductive outcomes, the pooled clinical pregnancy and live birth rates were 14.8% and 8.9%, respectively, suggesting a potential yet limited benefit for fertility restoration in this challenging patient population.

### 4.1. Critical Appraisal of Evidence from Randomized Controlled Trials

A pivotal strength of this analysis is the separate consideration of evidence from randomized controlled trials (RCTs), which provide the highest level of evidence for causal inference. The results from the two available RCTs, however, are inconclusive and highlight a critical discrepancy with uncontrolled studies. This stark discrepancy between the optimistic signals from lower-level, uncontrolled studies and the neutral findings from the highest level of evidence (RCTs) is a critical finding of this review. It underscores the danger of drawing clinical conclusions from non-randomized data and highlights the necessity of relying on controlled trials to determine true treatment efficacy.

Herlihy et al. (2024) [23] found no significant differences between PRP and control groups in mature oocyte yield, blastocyst formation, or live birth rates in women with poor ovarian response. Similarly, ovarian reserve markers showed no intergroup differences. In contrast, Barrenetxea et al. (2024) [22] reported a modest but significant increase in mature oocytes retrieved in the PRP group, yet no improvement in subsequent blastocyst development or live birth rates. Collectively, these RCTs suggest that any follicular response potentiated by PRP may not reliably translate into meaningful improvements in ultimate reproductive outcomes. This contrasts with the more optimistic signals from single-arm analyses, emphasizing the imperative to distinguish evidence by study design.

### 4.2. Sources of Heterogeneity and Methodological Considerations

The substantial heterogeneity observed stems from key methodological and clinical variations. We assessed statistical heterogeneity across studies using the Cochrane’s Q test and quantified its extent with the I^2^ statistic. Given the observed variability in study design, patient populations (including differences in diagnostic criteria such as Bologna and POSEIDON), PRP preparation protocols, and outcome measurement methods, a random-effects model was applied when substantial heterogeneity was detected (I^2^ > 50%). This approach allows for more conservative estimates by accounting for both within- and between-study variance.

Details of PRP protocols are listed in Table S3. PRP protocols. It is not difficult to find that significant differences occur, from centrifugation parameters, platelet concentration, and activation methods (e.g., calcium chloride vs. none) to injection techniques. For instance, Barrenetxea et al. used sequential centrifugation to obtain a specific plasma fraction, whereas Herlihy et al. employed a commercial kit—procedural diversity that directly influences growth factor bioavailability. Other factors, such as heterogeneity in population characteristics (e.g., diagnostic criteria, age, and baseline ovarian reserve), as well as short follow-up durations (typically <6 months), limit the assessment of effect durability and long-term safety.

Despite these methodological precautions, considerable heterogeneity persisted in several pooled analyses, reflecting the inherent diversity among the included studies. To further explore potential sources of heterogeneity, we plan to conduct subgroup analyses and meta-regression in future updates of this review, based on factors such as age, diagnostic criteria, PRP preparation techniques, and study design. These additional analyses will aim to clarify the conditions under which intraovarian PRP may exert more consistent effects and to inform the design of future standardized trials.

### 4.3. Clinical Relevance and the Translational Gap

The central finding of this review is the apparent translational gap between biomarker improvement and definitive clinical benefit. While improvements in AMH and AFC align with PRP’s proposed biological mechanisms (e.g., angiogenesis and anti-inflammatory effects via PDGF, TGF-β, and VEGF), the lack of consistent translation into higher live birth rates indicates that these effects may be insufficient to overcome the profound follicular depletion or oocyte quality deficits in advanced DOR. Therefore, improvements in ovarian reserve markers should not be equated with enhanced fertility. From a clinical perspective, intraovarian PRP remains an experimental intervention. Patients should be counseled that current high-level evidence does not consistently support its efficacy for improving live birth chances, despite promising signals from lower-level studies.

### 4.4. Strengths and Limitations

#### 4.4.1. Strengths

This meta-analysis possesses several methodological strengths. First, by restricting inclusion to prospective studies, we enhanced the temporal reliability of the observed associations. Second, a comprehensive and systematic literature search across multiple databases was conducted to minimize the risk of omission. Third, our study employed a novel stratified analytical approach, distinguishing evidence from uncontrolled and controlled designs, which provides a more nuanced and critical appraisal of the current evidence base. Finally, we systematically evaluated a comprehensive set of outcomes, encompassing both hormonal (AMH, FSH) and ultrasonographic (AFC) markers of ovarian reserve, as well as key reproductive endpoints.

#### 4.4.2. Limitations

Notwithstanding these strengths, our findings must be interpreted considering several constraints. The most significant limitation is the scarcity of randomized controlled trials (RCTs), which restricts the strength of causal inference. Furthermore, we observed moderate-to-high heterogeneity across studies, attributable to variations in PRP protocols, patient populations (e.g., the inclusion of women with DOR, POI, and POR, while increasing the breadth of the review, introduces clinical heterogeneity due to their distinct pathophysiologies. This limits the specificity of our findings, and future research with sufficient data should perform stratified analyses by diagnostic group), and outcome definitions, complicating the interpretation of pooled estimates. The reporting of standardized, clinically decisive reproductive outcomes (particularly live birth) was inconsistent and often based on short-term follow-up. Lastly, while statistical tests did not indicate significant publication bias, the possibility that small, negative studies remain unpublished cannot be entirely excluded.

## 5. Conclusions

In conclusion, intraovarian platelet-rich plasma administration may be associated with modest improvements in ovarian reserve markers such as AMH and AFC; however, current controlled evidence does not demonstrate a consistent benefit in clinically meaningful reproductive outcomes, including live birth. Given the low certainty of evidence, substantial methodological heterogeneity, and limited randomized data, PRP should be regarded as an experimental approach in women with diminished ovarian reserve. Well-designed, adequately powered randomized controlled trials with standardized PRP protocols and long-term follow-up are required before this intervention can be recommended for routine clinical practice.

## Figures and Tables

**Table 1:** Data extracted from the included literature. (* The “/” indicates the original literature didn’t provide this information).

Study (Year)	Country	Study Design	No. of Evaluable Patients/Grade	Age	BMI	Study Period	Treatment Protocol	Follow-Up (mo)	Type of Diagnostic Method During Follow-Up	Reported Outcomes
Aflatoonian et al. (2021) [18]	Iran	Before and after study	17 POR (Bologna), 9 POI (ESHRE)	POR: 35.47 ± 4.34 POI: 33.66 ± 4.84	POR: 25.68 ± 2.58 POI: 26.36 ± 3.62	2018–2020	PRP injection (5 POR patients once, others received the second PRP injection with a twofold increase 3 months later)	12	Hormonal measurement (ELISA), hCG + transvaginal ultrasonography	AMH, FSH, LH, E2, pregnancy outcome, menstrual restoration in POI group
Parvanov et al. (2022) [19]	Bulgaria	Before and after prospective study	66 POR (Bologna)	40.5 (Min–Max 34–46)	23.5 (Min–Max 19.0–27.0)	March 2021–September 2021	PRP injection (once between day 7 and day 11 of the menstrual cycle for two consecutive cycles) IVF (COS + oocyte retrieval + ICSI + embryo culture)	6	Serum AMH and FSH: electrochemiluminescent (ECLIA) Oocyte quality: protocol based on morphological assessment and enumeration of extracytoplasmic anomalies Rmbryo quality: morphological evaluation	AFC, AMH, FSH, fertilization rate, number and quality of oocytes and embryos
Cakiroglu et al. (2020) [20]	Turkey	Before and after prospective study	311 POI (ESHRE)	34.8 ± 4.3	/ *	2018–2019	PRP injection + IVF (COH was performed when antral follicles found, followed up to 6 months, if not treated with cyclic estrogen and progesterone)	/	AFC: transvaginal ultrasound serum AMH and FSH: immunoassays oocyte and embryo: during IVF pregnancy confirmation: serum β-HCG testing and ultrasound confirmation	AFC, AMH, FSH, fertilization rate, number and quality of oocytes and embryos
Devenutto et al. (2024) [15]	Argentina	Prospective cohort study	148 POR (Poseidon)	39.61 (33–44)	/	2021–2022	PRP injection (once, bilateral) + ART (≥3 antral follicles) + IVF	/	AFC: transvaginal ultrasound AMH, FSH, LH, E: blood test oocyte and embryo: during IVF Fertilization rate: the ratio of the number of 2 pronuclei embryos to the number of inseminated oocytes	AFC, AMH, FSH, LH, E fertilization rate, number and quality of oocytes and embryos
Barad et al. (2022) [21]	USA	Prospective cohort study	80 LFOR (group 1—54 DOR but still regular menstrual cycles; group 2—26 DOR but irregular menstrual cycles or amenorrhea)	Group 1: 44.2 ± 5 group 2: 44.1 ± 6.4	Group 1: 23.7 ± 4.3 group 2: 23.6 ± 4.1	2018–2021	PRP injection (once, bilateral) + IVF (ovulation initiated at 1 month after, median 2 cycles overall)	12	AFC: transvaginal ultrasound AMH, FSH, E: blood test oocyte and embryo: during IVF	AFC, AMH, FSH, E number and quality of oocytes and embryos
Barrenetxea et al. (2024) [22]	Spain	RCT	60 POR (17 POSEIDON 3 and 43 POSEIDON 4)	37.59 ± 0.41	22.91 ± 0.26	January 2021–December 2021	Experimental group: PRP injection Control group: placebo (saline) Both underwent ovarian stimulation	/	MII: counted after each of the three ovarian punctures blastocyst: PGT-A pregnancy rate: ultrasound	Number of mature oocytes, blastocyst pregnancy rate
Herlihy et al. (2024) [23]	USA	RCT	83POR	Experiment: 34.4 ± 2.8 Control: 34.7 ± 2.1	Experiment: 26.3 ± 4.1 Control: 24.6 ± 4.6	2020–2022	Experimental group (41): PRP injection 39 ovarian stimulation and oocyte retrieval Control group (42): no intervention 37 ovarian stimulation and oocyte retrieval	/	MII oocytes: during oocyte retrieval AFC: transvaginal ultrasonography Blastocyst and Euploid Blastocyst: trophectoderm biopsied and analyzed via PGTseq-A pregnancy outcomes: sustained implantation rate defined as fetal heartbeat confirmed at ~8 weeks gestational age	Primary: number of mature oocytes Secondary: AFC, AMH, number of blastocyst, or euploid blastocyst, pregnancy outcome
Hosseinisadat et al. (2023) [13]	Iran	Before and after study	22 POR (Bologna)	33.91 ± 6.58	/	2019–2020	ART (FSH + GnRH antagonist) + IVF (trigger ovulation with HCG and oocyte retrieval) + PRP injection	3	AMH: venous blood, ELISA AFC: vaginal ultrasound	AFC, AMH
Keikha et al. (2022) [24]	Iran	Quasi-experimental study	12 POR (Bologna 4)	40.04 ± 3.91	22.59 ± 9.76	August 2021–December 2021	IVF (GnRH antagonist + HMG + HCG and oocyte retrieval) + PRP injection (right side for experimental group)	/	AMH, FSH: ELISA AFC: transvaginal ultrasound oocyte and embryo: during oocyte retrieval	Primary: AFC, oocyte number, number and grade of embryos Secondary: AMH, FSH
Najafian et al. (2023) [25]	Iran	Quasi-experimental study	50 POR (POSEIDON grade 3 or 4)	39 (35–43)	25.3 ± 2.9	2021–2022	PRP injection (bilateral) + IVF	3	/	AFC, AMH, FSH, number of oocytes and embryos pregnancy outcomes
Navali et al. (2022) [26]	Iran	Before–after study	35 POR	40.68 ± 0.34	/	April 2021–May 2021	Ovarian stimulation (Letrozole + FSH + hMG + GnRH antagonsit + HCG) + PRP injection (bilateral)	2	Number of oocytes and embryos: transvaginal ultrasound FSH, LH, AMH, E: serum assays	FSH, LH, AMH, E, number of oocytes, blastocyst pregnancy rate
Petrtk et al. (2020) [27]	Ukraine	Original research article	38 LOR	31–45	/	/	PRP injection	12	FSH, LH, Estradiol, AMH: blood test pregnancy rate and oocyte: ultrasound, β-HCG subjective outcomes: patient-reported metrics	LH, FSH, AMH, E delivery of a baby, pregnancy rate, and own oocytes Subjective outcomes: regular menstruation self-satisfaction wish to have a baby retrieved
Davari tanha et al. (2022) [28]	Iran	Single-arm trial research	20 POR	41.80 ± 1.82	25.85 ± 3.16	2020–2021	PRP injection (bilateral) + ART (12 weeks after)	/	AFC: transvaginal ultrasound FSH, AMH, E: blood test oocyte number: during retrieval pregnancy outcomes: β-HCG two weeks after embryo transfer, ultrasound at six weeks	AFC, AMH, FSH, E, LH number of oocytes, pregnancy outcomes
Tehraninejad et al. (2023) [29]	Iran	Non-randomized clinical trial	56 POR (Bologna)	Experiment: 39.34 ± 3.88 Control: 40.81 ± 3.68	Experiment: 25.20 ± 4.43 Control: 25.20 ± 4.07	2020–2021	Antagonist regimen + HMG + Cetrotide + HCG 34 experiment: PRP injection (bilateral) 22 control: no intervention	12	AMH: ELISA oocyte and embryo quality: during IVF pregnancy: β-HCG	AMH, oocyte and embryo quality, pregnancy outcome
Cakiroglu et al. (2022) [14]	Turkey	Perspective observational study	510 POR (Poseidon)	40.3 ± 4.0	/	January 2020–December 2020	PRP injection + ovarian stimulation and retrieval + embryo formation + PGT-A	/	AFC: transvaginal ultrasound AMH and FSH: blood tests pregnancy outcomes: β-hCG 12 days post-embryo transfer transvaginal ultrasound cardiac activity at 12 weeks PGT-A: NGS oocytes identified via ICSI and denudation	AFC, AMH, FSH, number of oocytes, blastocysts, embryos, fertilization rate
Garavellas et al. (2023) [30]	Greece	Before–After Prospective Pilot Study	253 POR/POI (234 ≤ 50 y)	18–56	18.5–30	May 2018–December 2021	PRP injection	2	The quantification of FSH, LH, AMH, and E2 was performed using the chemiluminescent microparticle immunoassay using the Roche analyzer. (On the day 3 of the menstrual cycle)	FSH, LH, AMH and E2 For women with advanced ages (>48 years), the restoration and regularity of the menstrual cycle were additionally evaluated
Navarro et al. (2025) [31]	Venezuela	Prospective, randomized, observational, analytical study	30 DOR	41.20 ± 2.86 (38–46)	23–30	January 2024–September 2024	Group 1: autologous exosomes Group 2: PRP Group 3: saline solution	9	FSH, LH, Estradiol, AMH and AFC, MII oocytes, fertilization rate, frozen embryos and positive pregnancies (including β-HCG and 12-weeks)	FSH, LH, Estradiol, AMH and AFC, MII oocytes, fertilization rate, frozen embryos, and positive pregnancies
Stojkovska et al. (2019) [32]	Macedonia	Prospective pilot study	40 POR (Bologna)	Group A: 37.47 ± 3.87 Group B: 37.64 ± 3.20	Group A: 22.63 ± 3.81 Group B: 24.07 ± 5.01	June 2017–December 2018	Group A: PRP Group B:/ Both underwent IVF-ICSI	18	FSH, AMH, AFC, oocytes: ultrasonography and laboratory tests clinical pregnancy: positive serum hCG level, 2 weeks post-embryo transfer	FSH, AMH, estradiol and total AFC (no significant difference) MII oocytes, fertilization rate, clinical pregnancy rate, and live birth rate
Simavli et al. (2025) [33]	Turkey	Pre–post research	234 POR (Bologna)	33.2 ± 4.5	/	June 2019–May 2023	PRP injection	6	AFC, serum AMH and FSH were reassessed between days 2 and 4 before and after PRP therapy	FSH, AMH, E2, LH, AFC, pregnancy rate, and live birth rate

## Data Availability

In this systematic review and meta-analysis, all data were obtained from published studies.

## Supplementary Materials

The following supporting information can be downloaded at https://www.mdpi.com/article/10.3390/jcm15072482/s1. Table S1: Search strategies; Table S2: PRISMA checklist; Table S3: PRP protocols and quality assessment; Figure S1: Sensitivity analysis of birth rate; Figure S2: Sensitivity analysis of pregnancy rate; Figure S3: Funnel plot. Reference [35] is cited in Supplementary Materials.
