# Supplementary material for: Intraovarian Platelet-Rich Plasma for Women with Diminished Ovarian Reserve: A Systematic Review and Meta-Analysis
Source: J Clin Med. 2026 Mar 24;15(7):2482. doi: 10.3390/jcm15072482 (PMC13074068; doi:10.3390/jcm15072482)
Supplement: Supplementary file 1 [file jcm-15-02482-s001.zip › Table S1.search strategy.pdf]

PubMed	(((((((diminished ovarian reserve[MeSH Terms]) OR (poor ovarian response[MeSH Terms])) OR (premature ovarian insufficiency[Title/Abstract])) OR (DOR[Title/Abstract])) OR (POR[Title/Abstract])) OR (POI[Title/Abstract])) AND (((platelet-rich plasma[MeSH Terms]) OR (PRP[Title/Abstract])) OR (platelet rich plasma[Title/Abstract]))) AND ((((intraovarian[Title/Abstract]) OR (ovarian[Title/Abstract])) OR (injection[Title/Abstract])) OR (infusion[Title/Abstract]))
Web of Science	Refine results for ((((((diminished ovarian reserve) OR (poor ovarian response)) OR (premature ovarian insufficiency)) OR (DOR)) OR (POR)) OR (POI)) AND (((platelet-rich plasma) OR (PRP)) OR (platelet rich plasma))) and 2024 or 2025 or 2023 or 2022 or 2021 or 2020 or 2019 (Publication Years) and Article (Document Types) and English (Languages) and Reproductive Biology or Obstetrics Gynecology (Web of Science Categories) and 1.81.979 Assisted Reproduction or 1.81.1408 Fertility Preservation or 1.128.753 Gynecologic Oncology or 1.81.1272 Reproductive Immunology (Citation Topics Micro)
EMBASE	('poor ovarian response'/exp OR 'diminished ovarian reserve'/exp OR 'premature ovarian failure'/exp OR 'pof (premature ovarian failure)' OR 'ovarian failure, premature' OR 'premature ovarian failure' OR 'premature ovarian insufficiency' OR 'primary ovarian insufficiency') AND ('platelet-rich plasma cell'/exp OR 'prp' OR 'platelet rich plasma cell' OR 'platelet-rich plasma cell') AND ('fertility'/exp OR 'fecundity' OR 'fertility') AND ('randomized controlled trial'/exp OR 'controlled trial, randomized' OR 'randomised controlled study' OR 'randomised controlled trial' OR 'randomized controlled study' OR 'randomized controlled trial' OR 'trial, randomized controlled' OR 'cohort analysis'/exp OR 'analysis, cohort' OR 'cohort analysis' OR 'cohort fertility' OR 'cohort life cycle' OR 'cohort studies' OR 'cohort study' OR 'fertility, cohort')
Cochrane Central Register of Controlled Trials (CENTRAL)	("platelet-rich plasma" OR PRP) AND ("diminished ovarian reserve" OR "poor ovarian response" OR "premature ovarian insufficiency" OR DOR OR POR OR POI) in Title Abstract Keyword - (Word variations have been searched)

Scopus	((poi OR pof OR por OR dor) AND (prp OR platelet AND rich AND plasma)) AND<br>(randomized AND controlled AND trial) AND<br>PUBYEAR > 2018 AND PUBYEAR < 2026 AND (LIMIT-TO (SRCTYPE , "j"))<br>AND (LIMIT-TO (EXACTKEYWORD , "Human") OR<br>LIMIT-TO (EXACTKEYWORD , "Article") OR LIMIT-TO (EXACTKEYWORD , "Controlled Study")<br>OR LIMIT-TO (EXACTKEYWORD , "Female")) AND (LIMIT-TO (DOCTYPE , "ar"))<br>AND (LIMIT-TO (SUBJAREA , "MEDI")) AND (LIMIT-TO (LANGUAGE , "English"))
