# Supplementary figures and images for: Intraovarian Platelet-Rich Plasma for Women with Diminished Ovarian Reserve: A Systematic Review and Meta-Analysis
Source: J Clin Med. 2026 Mar 24;15(7):2482. doi: 10.3390/jcm15072482 (PMC13074068; doi:10.3390/jcm15072482)

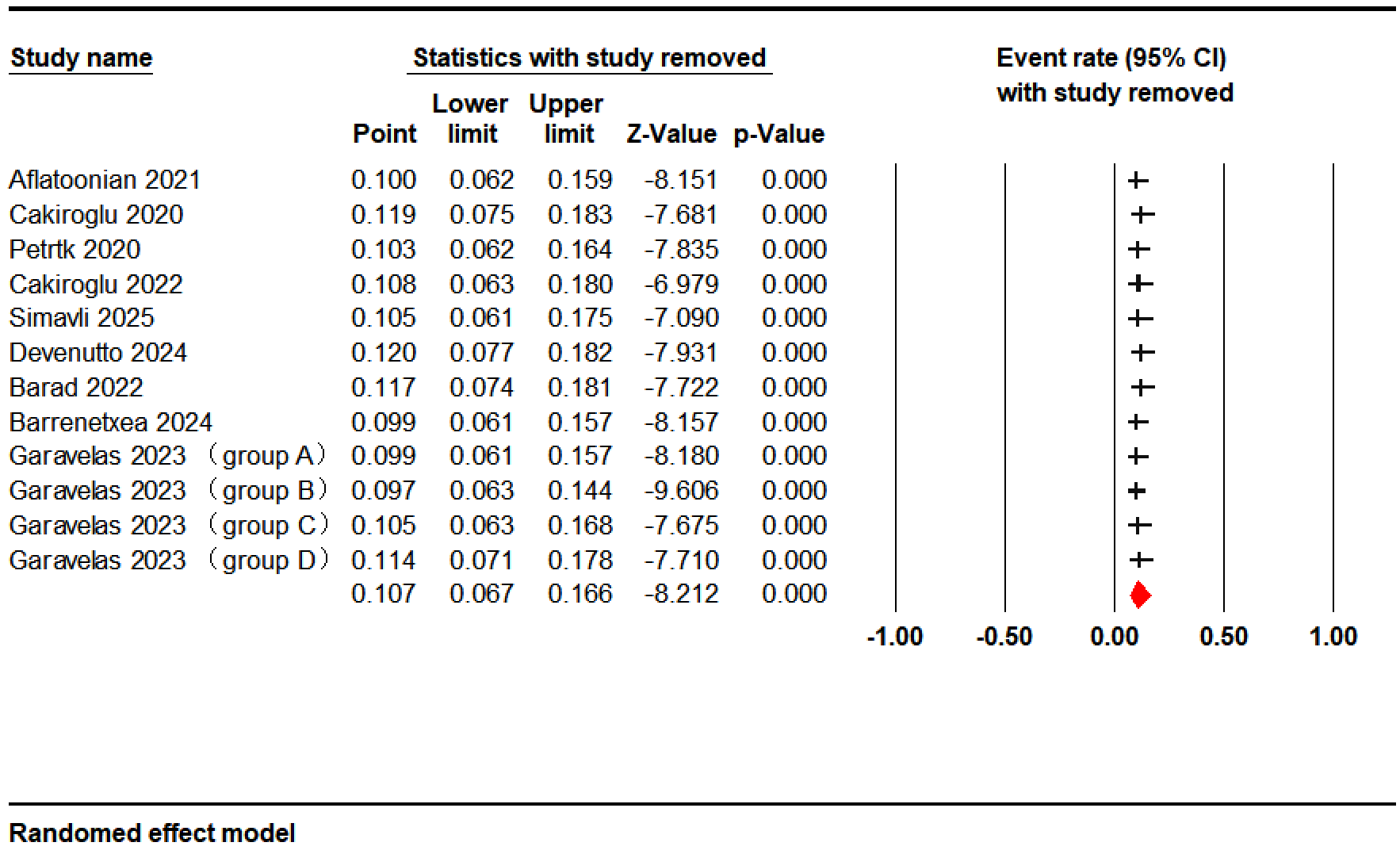

Supplement: Supplementary file 1 [file jcm-15-02482-s001.zip › Figure S1. leave-one-out birth rate.png]

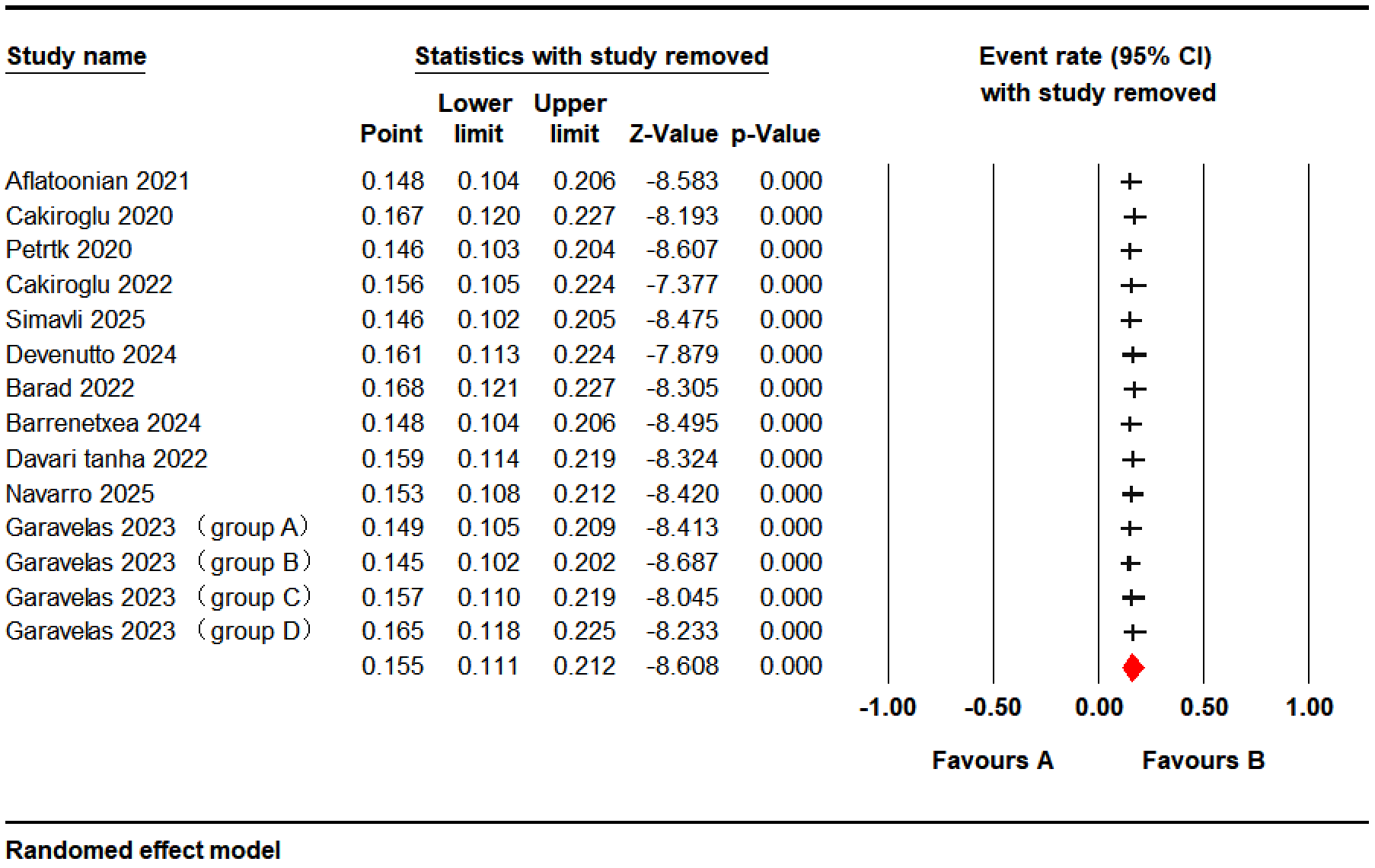

Supplement: Supplementary file 1 [file jcm-15-02482-s001.zip › Figure S2. leave-one-out pregnancy rate.png]

# Meta..... (ggplot2)

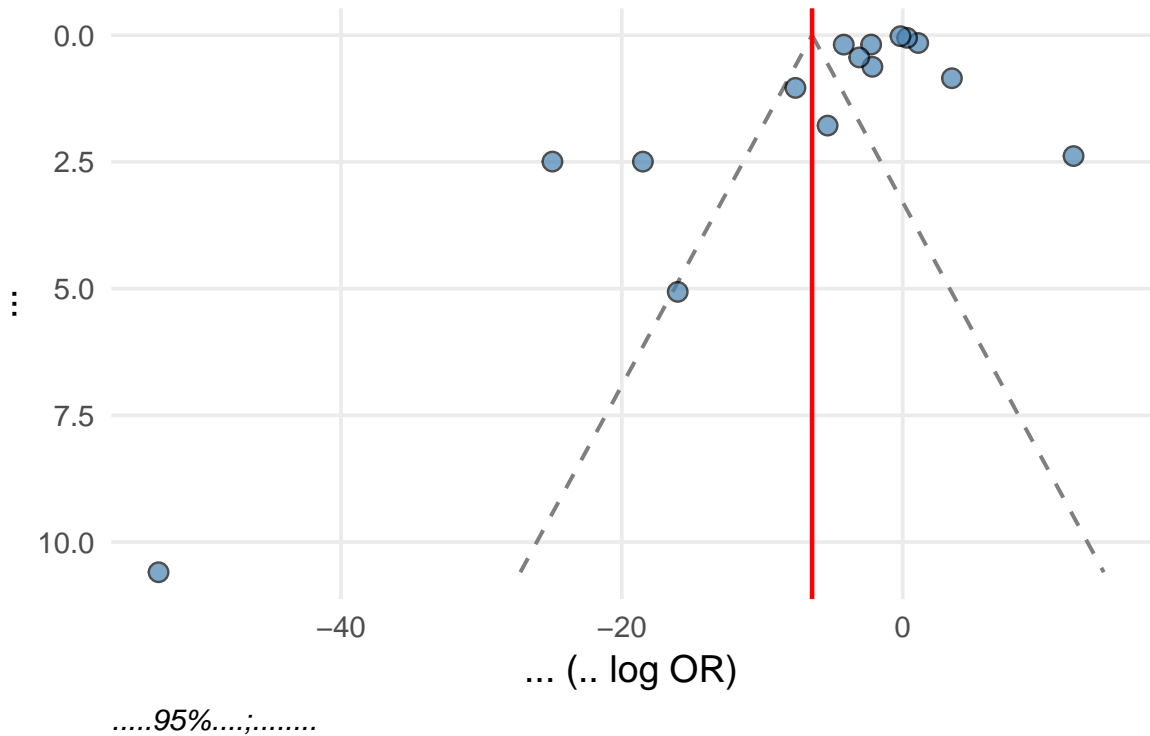

Supplement: Supplementary file 1 [file jcm-15-02482-s001.zip › Figure S3. funnel plot(FSH).pdf]
